# Lifestyle and environmental contributions to ovulatory dysfunction in women of polycystic ovary syndrome

**DOI:** 10.1186/s12902-020-0497-6

**Published:** 2020-01-30

**Authors:** Bingqian Zhang, Wei Zhou, Yuhua Shi, Jun Zhang, Linlin Cui, Zi-Jiang Chen

**Affiliations:** 1 Center for Reproductive Medicine, Shandong University, No.157 of Jingliu Street, Jinan, 250012 Shandong China; 2 National Research Center for Assisted Reproductive Technology and Reproductive Genetics, Shandong University, No.44 of Wenhua Street, Jinan, 250012 Shandong China; 3 Key laboratory of Reproductive Endocrinology of Ministry of Education, Shandong University, No.44 of Wenhua street, Jinan, 250012 Shandong China; 4 Shandong Provincial Clinical Medicine Research Center for Reproductive Health, Shandong University, No.157 of Jingliu Street, Jinan, 250012 Shandong China; 5 Ministry of Education-Shanghai Key Laboratory of Children’s Environmental Health, Xinhua Hospital, School of Medicine, Shanghai Jiao-Tong University, No.1665 of Kongjiang Street, Shanghai, 200092 China; 6 Shanghai Key Laboratory for Assisted Reproduction and Reproductive Genetics, No.845 of Lingshan Street, Shanghai, 200088 China; 7 Center for Reproductive Medicine, Ren Ji Hospital, School of Medicine, Shanghai Jiao Tong University, No.845 of Lingshan Street, Shanghai, 200088 China

**Keywords:** PCOS, Environmental factors, Lifestyle, Ovulatory dysfunction

## Abstract

**Background:**

Polycystic ovary syndrome (PCOS) is the most common reason of anovulatory infertility. Environmental factor is one of the main causes of PCOS, but its contribution to ovulatory dysfunction in PCOS remains unknown.

**Methods:**

A total of 2217 infertile women diagnosed as PCOS according to Rotterdam criteria were recruited, including 1979 women with oligo-anovulation (OA group) and 238 women with normal -anovulation (non OA group). Besides, 279 healthy control women of reproductive age were enrolled as controls.

**Results:**

Frequencies of snoring (PCOS-OA group, PCOS-non-OA group, control group: 29.30% vs 18.10% vs 11.50%, *P* < 0.01), smoking (37.70% vs 28.10% vs 12.20%, *P* < 0.01), plastic tableware usage (38.30% vs 28.10% vs 25.40%, *P* < 0.01) and indoor decoration (32.10% vs 24.80% vs 16.80%, *P* < 0.01) were highest in PCOS-OA group. After adjusted for multivariable, difference remained significant between PCOS-OA group and the other two groups. PCOS-OA women preferred a meat favorable diet compared to PCOS-non-OA group (54.60% vs 41.30%, *P* < 0.01). There was no difference between three groups in exercise, frequency of insomnia, and alcohol consumption.

**Conclusions:**

Smoking, snoring, hyper-caloric diet, plastic tableware usage and indoor decoration were found to be associated with an increased risk for ovulatory dysfunction in women suffering from PCOS.

## Background

Polycystic ovary syndrome (PCOS) is a heterogeneous endocrinopathy with a prevalence of approximately 5.5–19.9% [1]. It is characterized by irregular cycles, clinical or biochemical hyperandrogenism, and polycystic ovarian morphology (PCOM). Moreover, it is the most common cause of chronic anovulatory infertility [2]. As reported previously, 90.30–95.28% of PCOS women diagnosed by Rotterdam criteria were characterized with ovulatory dysfunction [3, 4]. Additionally, oligo−/anovulatory (OA) subjects showed more severe hormonal and metabolic derangements compared to PCOS women with normal ovulation [4, 5]. According to Chinese diagnostic criteria, ovulatory dysfunction was even considered as necessity for the diagnosis of the syndrome [6]. However, the pathogenesis has not been fully understood.

Environmental factors and genetic variants are both well accepted as main etiologic factors of PCOS. Several susceptibility genes have been reported via candidate gene screening and GWAS [7–9]. However, environmental components, being another key factor in the pathophysiology, have not been well documented. It was shown that environmental endocrine disruptors (EEDs) could perturb the hormonal regulation of the hypothalamic pituitary ovarian axis. Hence, EEDs are supposed to act as steroid-agonists and/or antagonists [10–12]. Previous studies also indicated a correlation between PCOS and contact history of EEDs, as well as changes of environment and lifestyle [13, 14]. However, the exact effect of environmental factors on ovulatory dysfunction in PCOS women remains unclear.

In our study, lifestyles and EED exposure of PCOS women with and without OA were analyzed in order to determine the association between environmental factors and ovulatory dysfunction of PCOS.

## Methods

### Participants

A total of 2217 PCOS women and 279 non-PCOS women were recruited from April 2006 to December 2009 in the Center for Reproductive Medicine, Shandong Provincial Hospital Affiliated to Shandong University. Among PCOS patients, patients with oligo−/anovulation were included in PCOS-OA group (*N* = 1979). Patients with normal menses but with hyperandrogenism and PCOM were included in the PCOS-non-OA group (*N* = 238). All of the participants were recruited from a prospective cohort study including women undergoing assisted reproductive technology treatment. The non-PCOS women who have previously participated in another studies were recruited as healthy controls [15, 16].

All PCOS women were diagnosed according to Rotterdam criteria with any two of following phenotypes: 1) OA, 2) hyperandrogenism, 3) PCOM [17, 18]. Moreover, absence of other causes of ovulatory dysfunction and hyperandrogenism including 21-hydroxylase deficiency, congenital adrenal hyperplasias, androgen-secreting tumors, Cushing’s syndrome, hyperprolactinemia, and thyroid disease were excluded. OA was defined as the duration of menstrual cycle more than 35 days in length or a history of less than 8 spontaneous hemorrhagic episodes per year [19]. Hyperandrogenism was determined when there was either biochemical hyperandrogenemia (total testosterone levels in early follicular phase≥60 ng/dl) or hirsutism (modified Ferriman-Gallwey score ≥ 6). Trans-vaginal ultrasonic examination was performed, and PCOM was considered when 12 or more follicles with a diameter of 2–9 mm were found in at least one ovary and/or the ovarian volume was over 10 ml [17, 18]. All women in control group were undergoing treatment for tubal obstruction or male factor induced infertility. All of them had normal menstrual cycles.

### Clinical and biochemical measurements

Information on age, height, weight, and medical history were recorded during clinical examination. Menstruation information of women in PCOS non-OA group were recorded without drug usage. Body mass index (BMI) was calculated as weight (kg)/height (m^2^). Questionnaire including social information, life style and environmental contact history in daily life were completed by the same trained interviewer. Lifestyle referred to insomnia, snoring, meat favorable diet, smoking, alcohol consumption, tea drinking, and exercise duration. Assessment of environmental exposure includedthe usage of plastic tableware, indoor decoration, air freshener, and cooking oil fume. Definitions of those items were presented in Table 2.

Fasting blood sampling was collected during early follicular phase. The parameters including follicular stimulating hormone (FSH), luteinizing hormone (LH), total testosterone (TT), and prolactin (PRL) using chemiluminescence immunization. The intra- and inter-assay variation coefficients of variation are < 10%.

### Statistical analysis

Statistical analysis was performed using Statistical Package for the Social Sciences for Windows (version 22.0; SPSS Inc., Chicago, IL, USA). Normality of data was assessed by Q-Q plot. Continuous normal distributed variables were presented as mean ± SD. One-way ANOVA test and t test were undertaken for continuous normal distributed variables. Х^2^ analysis were undertaken for categorical variables. Multivariate logistic regression was performed to adjust potential confounders, such as age and BMI. Statistical significance was set at level of 0.05.

## Results

**Fig. 1:**
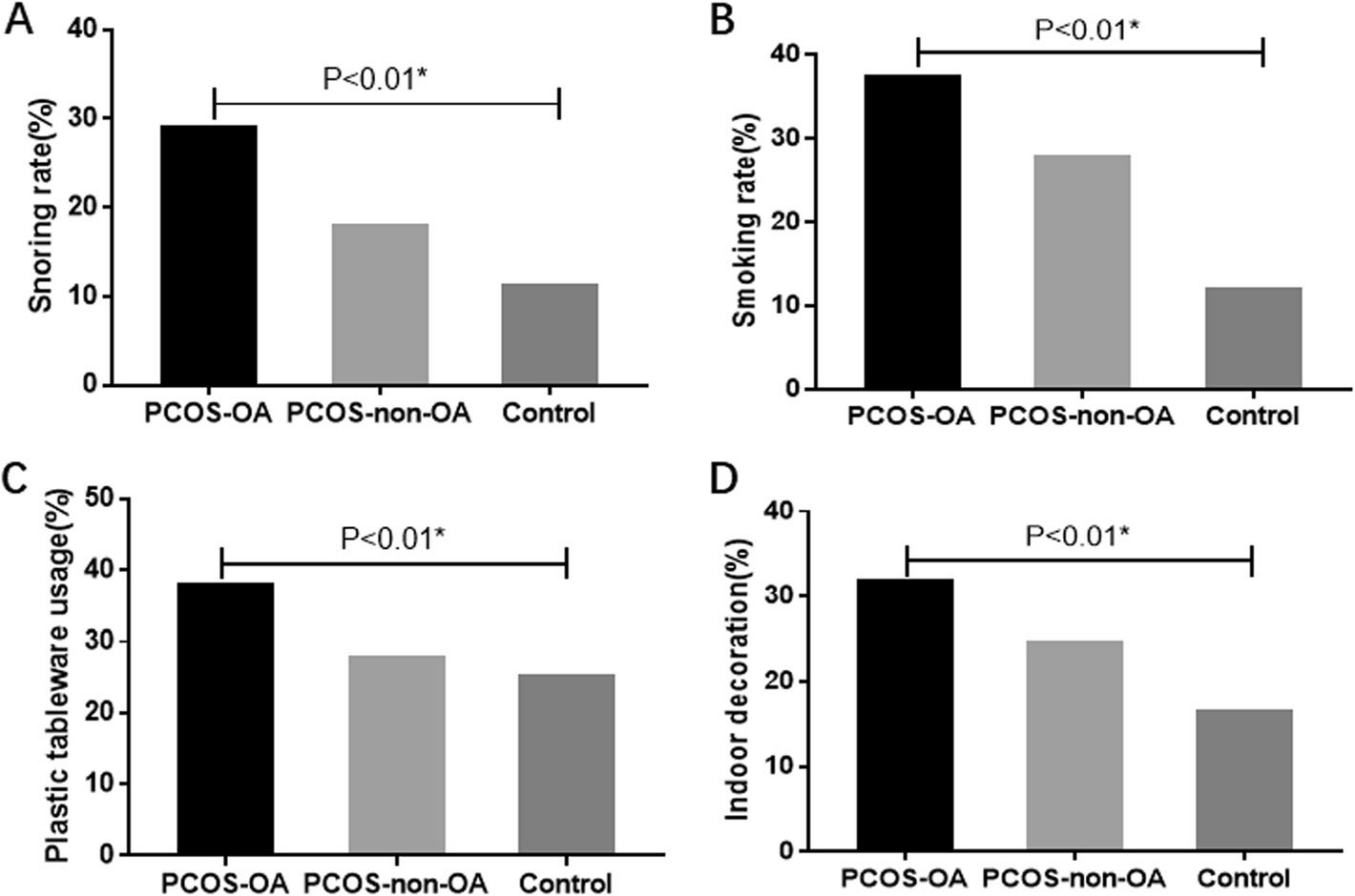
Lifestyle and environmental presence in oligo−/anovulation PCOS, eumenorrheic PCOS and control women. **a** Presence of snoring **b** Presence of smoking **c** Presence of plastic tableware usage **d** Presence of indoor decoration. OA:oligo−/anovulation. *: significance was set at level of 0.017(0.05/3).

Basic characters were shown in Table 1. PCOS women were older (PCOS-OA group, PCOS-non-OA group, Control group: 31.11 ± 3.71 vs 31.56 ± 3.49 vs 29.81 ± 3.77 yrs., P<0.01) and had a higher BMI (24.90 ± 4.09 vs 25.31 ± 4.39 vs 22.93 ± 3.86 kg/m2, P<0.01) compared to controls. Results of univariate analysis were shown in Table 2. Frequency of snoring (29.30% vs 18.10% vs11.50%, *P* < 0.01) and smoking (37.70% vs 28.10% vs 12.20%, *P* < 0.01) were different among three groups and were highest in the PCOS-OA group (Table 2, Fig. 1). After multivariate adjustment of age and BMI, these differences remained statistically significant except for the comparison of snoring between PCOS-non-OA and control group (Table 3). PCOS women, both with OA and without OA, preferred to drink tea more often than control women, even after adjustment (Table 3). Diet composition was only recorded in PCOS women. The comparison showed that PCOS-OA women had higher rate of meat favorable diet than their non-OA counterparts (54.60% vs 41.30%, *P* < 0.01; 1.69(1.28, 2.23), *P* < 0.01 after adjustment). No differences were found in exercise duration (*P* = 0.08), frequency of insomnia (*P* = 0.18), alcohol consumption (*P* = 0.92) amongst the three groups.

**Table 1:** Basic characteristic in oligo−/anovulation PCOS women, eumenorrheic PCOS women and control women

	PCOS-OA	PCOS-non-OA	Con	*P*
No.	1979	238	279	
Age (yrs)	31.11 ± 3.71	31.56 ± 3.49	29.81 ± 3.77	< 0.01^a^
BMI (kg/m2)	24.90 ± 4.09	25.31 ± 4.39	22.93 ± 3.86	< 0.01^a^
Total AFC	27.63 ± 8.06	27.68 ± 9.23	14.72 ± 5.44	< 0.01^a^
LH/FSH	1.50 ± 0.81	1.58 ± 0.85	0.79 ± 0.30	< 0.01^a^
T (ng/dl)	80.14 ± 18.82	67.79 ± 23.46	23.78 ± 12.83	< 0.01^a^
E2(pg/ml)	45.73 ± 19.36	45.22 ± 19.13	34.87 ± 25.42	< 0.01^a^

Data was presented as Mean ± SD for normality distributions. One-way anova and t test were used in continuous variables

*Abbreviation*: *BMI* Body mass index

^a^significance was set at level of 0.05

**Table 2:** Univariate Comparison in oligo−/anovulation PCOS women, eumenorrheic PCOS women and control women

		PCOS-OA	PCOS-non-OA	Control	*P*
No.		1979	238	279	
Meat favorable diet %(N)		54.60(1061)	41.30(95)	NA	<0.01^a^
Exercise duration per week %(N)					0.08
Less than 10 h		64.90(1274)	57.10(133)	65.60(181)	
10–20 h (including 10 h)		23.80(468)	26.60(62)	24.60(68)	
More than 20 h (including 20 h)		11.30(221)	16.30(38)	9.80(27)	
Insomnia%(N)					0.18
Rare		81.40(1596)	87.60(205)	83.10(231)	
1-3times/month		11.00(215)	6.40(15)	9.40(26)	
Every week		7.60(149)	6.00(14)	7.60(21)	
Snoring %(N)		29.30(579)	18.10(43)	11.50(32)	<0.01^a^
Smoking %(N)		37.70(734)	28.10(66)	12.20(34)	<0.01^a^
Alcohol consumption %(N)		7.10(141)	6.70(16)	6.50(18)	0.92
Tea drinking (%(N)		14.10(273)	15.80(37)	7.00(19)	< 0.01^a^
Plastic tableware usage %(N)		38.30(750)	28.10(66)	25.40(71)	< 0.01^a^
Indoor decoration at home or workplace %(N)		32.10(635)	24.80(59)	16.80(47)	< 0.01^a^
Air freshener usage %(N)		15.60(307)	9.30(22)	12.90(36)	0.02^a^
Cooking oil fume contact %(N)		53.00(1026)	43.00(101)	32.30(90)	< 0.01^a^

Data was presented as Mean ± SD and median (interquartile range) for normality and non-normality distributions respectively. One-way anova was used in continuous variables, while Chi square test was used in categorical variables

Smoke: Active smoking was defined as a person who currently smoke at least 1 cigarette per 3 days. Passive smoking was defined as a person who inhale smoke constantly from others in work or living area

Alcohol drinking: once a week for at least 6 months; Tea drinking: one cup per day for at least 6 months; Plastic tableware usage: 1 time per day for at least 6 months; Indoor decoration at home or workplace: indoor painting performed or large furniture moved within two years before incidence of dysmenorrhea ; Air freshener usage: 3 times per week for at least 6 months; Cooking oil fume contact: Cooking oil fume contact was defined as women who cooked at home or workplace at least once per 3 days

^a^significance was set at level of 0.05

**Table 3:** Multivariate Comparison in oligo−/anovulation PCOS women, eumenorrheic PCOS women and control women

	PCOS-OA vs PCOS-non-OA		PCOS-OA vs Control		PCOS-non-OA vs Control	
	OR(95%CI)	*P*	OR(95%CI)	*P*	OR(95%CI)	*P*
Meat favorable diet %(N) ^a^	1.69(1.28,2.23)	< 0.01^b^	NA	NA	NA	NA
Snoring %(N) ^a^	2.17(1.51,3.12)	< 0.01^b^	2.25(1.49,3.40)	< 0.01^b^	0.96(0.56,1.65)	0.89
Smoking %(N) ^a^	1.52(1.13,2.05)	< 0.01^b^	3.69(2.47,5.52)	< 0.01^b^	2.39(1.47,3.89)	< 0.01^b^
Tea drinking %(N) ^a^	0.90(0.62,1.31)	0.58	1.88(1.12,3.14)	0.02^b^	2.11(1.14,3.89)	0.02^b^
Plastic tableware usage %(N) ^a^	1.55(1.14,2.09)	< 0.01^b^	2.00(1.47,2.72)	< 0.01^b^	1.31(0.87,1.98)	0.19
Indoor decoration at home or workplace %(N) ^a^	1.40(1.03,1.91)	0.03^b^	2.53(1.78,3.57)	< 0.01^b^	1.86(1.19,2.92)	< 0.01^b^
Air freshener usage %(N) ^a^	1.77(1.12,2.79)	0.01^b^	1.27(0.86,1.88)	0.23	0.69(0.38,1.24)	0.21
Cooking oil fume contact %(N) ^a^	1.48(1.13,1.95)	< 0.01^b^	1.10(0.84,1.46)	0.49	0.73(0.50,1.06)	0.10

*Abbreviation*: *BMI* Body mass index

^a^Multivariate logistic regression was performed for categorical variables. Adjusted parameters included age, BMI, and groups

^b^significance was set at level of 0.05

The higher usage of plastic tableware (38.30% vs 28.10% vs 25.40%, *P* < 0.01) and indoor decoration (32.10% vs 24.80% vs 16.80%, *P* < 0.01) were found in PCOS-OA women compared with either PCOS-non-OA or control women (Table 2, Fig. 1). Similar trends were found after adjusting age and BMI. Furthermore, indoor decoration showed a gradient trend among three groups [OR: 1.40, 95%CI: 1.03–1.91, *P* = 0.03 for PCOS-OA vs PCOS-non-OA, OR: 2.53, .95%CI: 1.78 to 3.57), *P* < 0.01 for PCOS-OA vs Control, OR: 1.86, 95%CI: 1.19 to 2.92, *P* < 0.01 PCOS-non-OA vs control, Table 3]. Air refresher usage (15.60% vs 9.30% vs 12.90%, *P* < 0.01), and cooking oil fume contact (53.00% vs 43.00% vs 32.30%, *P* < 0.01) were more frequent in PCOS-OA women compared to their non-OA counterparts. But these differences were non-significant after adjustment (Table 3). We also compared education, occupation, sleep duration, source of drinking water, pesticide free of fruits and vegetables, and microwave usage among three groups, but found no difference (data not shown).

## Discussion

In the present study, smoking, snoring, meat favorable diet, and usage of plastic tableware and indoor decoration were found to be associated with oligo or anovulation in PCOS women. Moreover, smoking and indoor decoration contact seemed to have a dose-dependent effect. Tea drinking was positively associated with PCOS but not ovulatory dysfunction. These factors indicated that lifestyle and environmental endocrine disruptors may associated with the pathophysiology of PCOS.

Smoking was found to be associated with ovulatory dysfunction in a dose-dependent way in our study. Several studies have observed that smoke toxicants can disrupt folliculogenesis, leading to premature luteinization of preovulatory follicle. These toxicants can also decrease oocytes maturation and ultimately accelerated depletion of the primordial follicle pool [20, 21] . However, other studies found there was no correlation between smoking and OA in PCOS women [22, 23]. The main difference between previous studies and our study is the definition of smoking. In our study, exposure to smoking included both active and passive smoking, whereas other studies only included active smoking.

Snoring was found to be another lifestyle indicator associated with OA in PCOS. It is one of the earliest symptoms of obstructive sleep apnea (OSA), which is a complex disorder characterized by repetitive collapse of the pharyngeal airway during sleep [24]. Previous studies have reported that PCOS is associated with a reduction in REM sleep stage time and increased risk for OSA [25–27]. Alerted reproductive hormone secretion (i.e. high androgen and low estrogen levels) might contribute to the higher prevalence of OSA [28]. Furthermore, it was indicated that low estradiol-to-testosterone ratio was associated with chronic oligo-anovulatory cycles in PCOS [29].

The present study also confirmed the correlation of meat favorable diet and the risk of ovulatory dysfunction, which was consisted with findings in animal studies. Previous studies showed that a decrease of primordial and Graafian follicles in high-fat feeding rat [30]. Insulin resistance (IR) was supposed as one of the potential mechanisms. Studies in both animal and human beings demonstrated that hyper-caloric diet will induce IR and β-cell dysfunction [31, 32]. IR was found to interrupt follicle development through inhibiting hypothalamic positive feedback to estradiol (E2). Besides, the direct lesion on ovulation through kisspeptin down-expression and granulosa cell apoptosis were also indicated as underlying mechanism in high-fat diet feeding mice [33, 34].

Nowadays, more and more people pay attention to the impact of EEDs on female reproductive health. We found plastic tableware usage and indoor decoration were associated with PCOS ovulatory dysfunction in the present study. It was demonstrated that Bisphenol A (BPA), the main component of plastic containers, had a positive association with endocrine disturbances in PCOS [35, 36]. It acted as a potent binder of sex hormone-binding globulin [37] and had a bidirectional interaction effect with androgens [38, 39], which may result in perturbed ovarian steroidogenesis and folliculogenesis. However, recent studies indicated that the BPA exposure didn’t alter ovulation in mice [40, 41]. Hence, more in vivo studies were needed to replicate the association of BPA exposure and ovulatory dysfunction, and to elucidate the mechanism. Except for BPA, the plasticizer and its substitute diisononyl phthalate (DiNP), were other EED’s found in many consumers. DEHP or DiNP exposure was demonstrated to accelerate primordial follicle recruitment by up-regulating the PI3K pathway, and lead to prolonged estrous cyclicity and subfertility in female mouse. The pathological effect could last even long after cessation of exposure [42, 43] .

We also found a positive association between indoor decoration and PCOS ovulatory dysfunction. However, there were limited studies focusing on the association between indoor decoration and PCOS [13]. It was found that organic solvents, the most important constituents for indoor decoration, had a negative influence on glucose metabolism impairment [44, 45]. Hundreds of chemicals were detected in organic solvents that could activate tumor necrosis factor α (TNFα), one of the most famous proinflammatory cytokines, which resulted excessive hepatic glucose formation, inhibited muscular glucose uptake, and impaired insulin sensitivity [46, 47] through different pathways [47–49]. There was a definite association between insulin resistance and interrupted follicle development. This may explain the correlation between indoor decoration and PCOS.

The strength of the present study was that it has large samples. Besides, it evaluated the association of lifestyle and exposure to environmental pollutants with ovulatory dysfunction in PCOS women systemically, which would provide important indication on next-step etiological study of impacts of environment factors on ovulation dysfunction and PCOS. However, it still had several limitations. Firstly, as a self-report questionnaire, social desirability cannot be eliminated. Secondly, only a minority of Chinese women smoke due to cultural habits. Thus, the sample size is too small to conduct further subgroup analysis to examine the contribution of active or passive smoking. Besides, the exact caloric intake per day was not recorded in our data. Comparison of diet composition only cannot provide further does-dependent evidence. Specific design studies involving caloric intake were needed to explore the effect of diet on oligo−/anovulatory in PCOS.

## Conclusions

Ovulatory dysfunction in PCOS is related to unhealthy lifestyle and environmental pollutants exposure. Hence, lifestyle modification as the first-line therapy for PCOS especially women with OA should be promoted more vigorously.

## Data Availability

The datasets are available from the corresponding author on reasonable request.

## Abbreviations

BMI: Body mass index
BPA: Bisphenol A
DEHP: Di(2-ethylhexyl) phthalate
DiNP: Diisononyl phthalate
EEDs: Environmental endocrine disruptors
FSH: Follicular stimulating hormone
LH: Luteinizing hormone
OA: Oligo-anovulation
PCOM: Polycystic ovarian morphology
PCOS: Polycystic ovary syndrome
PRL: Prolactin
TNFα: Tumor necrosis factor α
TT: Total testosterone
